# Piscine orthoreovirus (PRV) infects Atlantic salmon erythrocytes

**DOI:** 10.1186/1297-9716-45-35

**Published:** 2014-04-03

**Authors:** Øystein Wessel Finstad, Maria Krudtaa Dahle, Tone Hæg Lindholm, Ingvild Berg Nyman, Marie Løvoll, Christian Wallace, Christel Moræus Olsen, Anne K Storset, Espen Rimstad

**Affiliations:** 1 Department of Food Safety and Infection Biology, Norwegian University of Life Sciences, Oslo, Norway; 2 Section of Immunology, Norwegian Veterinary Institute, Oslo, Norway; 3 VESO Vikan, Vikan, Namsos, Norway

## Abstract

Piscine orthoreovirus (PRV) belongs to the *Reoviridae* family and is the only known fish virus related to the *Orthoreovirus* genus. The virus is the causative agent of heart and skeletal muscle inflammation (HSMI), an emerging disease in farmed Atlantic salmon (*Salmo salar* L.). PRV is ubiquitous in farmed Atlantic salmon and high loads of PRV in the heart are consistent findings in HSMI. The mechanism by which PRV infection causes disease remains largely unknown. In this study we investigated the presence of PRV in blood and erythrocytes using an experimental cohabitation challenge model. We found that in the early phases of infection, the PRV loads in blood were significantly higher than in any other organ. Most virus was found in the erythrocyte fraction, and in individual fish more than 50% of erythrocytes were PRV-positive, as determined by flow cytometry. PRV was condensed into large cytoplasmic inclusions resembling viral factories, as demonstrated by immunofluorescence and confocal microscopy. By electron microscopy we showed that these inclusions contained reovirus-like particles. The PRV particles and inclusions also had a striking resemblance to previously reported viral inclusions described as Erythrocytic inclusion body syndrome (EIBS). We conclude that the erythrocyte is a major target cell for PRV infection. These findings provide new information about HSMI pathogenesis, and show that PRV is an important factor of viral erythrocytic inclusions.

## Introduction

Piscine orthoreovirus (PRV) is the causative agent of heart and skeletal muscle inflammation (HSMI), an important emerging disease in farmed Atlantic salmon (*Salmo salar* L*.*) [1,2]. HSMI is characterized by epi-, endo- and myocarditis, with infiltration of mononuclear CD8 positive cells, as well as myositis in red skeletal muscle [3,4]. The mechanism by which PRV infection causes disease remains largely unknown. PRV has a segmented, double-stranded RNA (dsRNA) genome and belongs to the *Reoviridae* family [2]. Other fish reoviruses are grouped in the genus *Aquareovirus*, but phylogenetic analysis indicates that PRV branches off the common root of the genera *Orthoreovirus* and *Aquareovirus,* although it clusters more closely with the orthoreoviruses [5,6]. PRV has 10 genomic segments, as do the orthoreoviruses, but the overall amino acid identity between the homologous proteins is very low, particularly for the surface-exposed and non-structural proteins. However, several amino acid motifs central to protein function are conserved for orthoreoviruses and PRV [6]. Unlike most orthoreoviruses, but similar to the mammalian orthoreoviruses (MRV), PRV is non-fusogenic [5]. This unique taxonomic placement of a fish virus within the *Reoviridae* family makes PRV particularly interesting. One genogroup and two sub-groups have been suggested after genomic analysis of PRV, but no specific sequence motifs have been found to be correlated with virulence [7,8]. The lack of an in-vitro cultivation system has restricted the progress of the study of PRV.

PRV is ubiquitous in farmed Atlantic salmon. Although high loads of PRV in the heart are a consistent finding in HSMI outbreaks, the virus can be detected at low levels in fish throughout the production cycle [8,9]. PRV has also been detected in farmed Atlantic salmon in Canada and Chile, farmed steelhead trout (*Oncorhynchus mykiss*), wild chum salmon (*O. keta*) and cutthrout trout (*O. clarkii*) in Canada [7] and in wild Atlantic salmon and brown trout (*Salmo trutta* L.) in Norway [10]. MRV infections are also ubiquitous in mammals. Although natural infections are generally considered benign, MRV Type 3 Dearing (T3D) has been used to induce myocarditis experimentally in mice [11]. Avian orthoreovirus (ARV) infections in chicken and turkey are associated with several disease conditions [12-14]. In both poultry and aquaculture farming, large numbers of animals are kept confined at high densities. These conditions may be stressful and cause depression of immune responses, while simultaneously facilitating transmission of infectious agents.

HSMI was first described in Norway in 1999 [1]. Since then the number of outbreaks has increased and in 2012 there were 142 registered outbreaks [15]. The disease has also been reported in Scotland [16]. HSMI is mainly observed during the seawater grow-out phase of the fish, with morbidity close to 100% in affected cages, while cumulative mortality varies from negligible to 20% [17]. Typical gross pathologic changes in affected fish include signs of circulatory disturbance; pale heart, ascites, yellow liver, swollen spleen and petechiae in perivascular fat.

Diagnosis of HSMI is currently based on typical histopathological findings in heart and red skeletal muscle [3,4]. Both the viral load in the heart, as measured by reverse transcription quantitative PCR (RT-qPCR) [4], and the presence of viral proteins in cardiomyocytes, as demonstrated by immunohistochemistry [18], correlate with the development of heart lesions, and indicate that PRV replicates in heart tissue. However, relatively high PRV loads have been detected in both farmed and wild salmon without presence of histopathological changes [8,10].

PRV has been detected in the spleen and head kidney at higher loads than those of the heart [19], and inoculates originating from heart, liver, kidney/spleen and blood plasma from diseased fish have been used to reproduce disease [20]. The pathogenesis of PRV infection in salmon is largely unknown and possible consequences of PRV infection not related to HSMI should be further investigated. Interestingly, when immunohistochemistry was performed on heart sections from experimentally PRV-challenged fish, circulating cells were found to be PRV positive prior to detection of PRV in cardiomyocytes [18]. The PRV-positive blood cells were located in the cardiac lumen and in blood vessels and included both erythrocytes and leukocyte-like cells. Unfortunately, the available material of that experiment was not suitable for further characterization of these cells. The presence of viremia in PRV infection has not been studied.

A previous study of field material from four separate HSMI outbreaks reported several different virus like particles (VLP) by electron microscopy (EM) in erythrocytes, and also in macrophages in the head kidney. However, no known virus species have been linked to these findings [21]. One of these VLPs had similar morphology to those detected in erythrocytes of many species of salmonid fish; collectively called erythrocytic inclusion body syndrome (EIBS) [22-26]. No specific virus has been characterized from the EIBS-inclusions and whether one or several viral species are involved has not been documented.

PRV encodes a homologous protein of the MRV outer-fiber protein (σ1) that is present in most members of genus *Orthoreovirus* and is known to be the cell attachment protein [5,6]*.* MRV bind erythrocytes by the σ1 protein [27-30], and amino acid residues central for this binding are partly conserved in the PRV σ1 protein [5,6]. In contrast to mammalian erythrocytes, piscine red blood cells (RBC) are nucleated and thus have the potential to support viral replication [31].

We hypothesized that there is a cell-associated viremia in PRV infection that is of importance for the pathogenesis. In two consecutive PRV challenge experiments based on cohabitant transfer of virus, we assessed viral loads in blood, plasma and erythrocytes in the different phases of infection.

## Materials and methods

### Experimental fish and rearing conditions

Two consecutive PRV challenge experiments were conducted at VESO Vikan aquatic research facility, Vikan, Norway. Both trials were performed by cohabitation challenge, using unvaccinated, seawater-adapted Atlantic salmon that were confirmed free of known salmon pathogens. The fish were kept in tanks supplied with filtered and UV-radiated 30.5-34.6% salinity seawater, 12 °C ± 0.54 °C and with a 24 h light/0 h dark regime. The fish were acclimatized for 2 weeks prior to challenge, fed according to standard procedures and anesthetized by bath immersion (2–5 min) in benzocaine chloride (0.5 g/10 L water) (Apotekproduksjon AS; Oslo, Norway) before handling. Fish were killed using concentrated benzocaine chloride (1 g/5 L water) for 5 min. Control samples from 6 fish were collected before initiation of the experiments. The experiments were approved by the Norwegian Animal Research Authority.

### Challenge experiment # 1

50 shedders and 50 cohabitants with an initial average weight of 48 g were used. The inoculum originated from a field outbreak of HSMI as determined by histopathological examination and RT-qPCR that showed high loads of PRV. Four fish from the outbreak, with a mean Ct-value 25.6 for PRV in the heart, were used. The fish were confirmed free of infectious salmon anemia virus (ISAV), salmonid alphavirus (SAV), piscine myocarditis virus (PMCV) and infectious pancreatic necrosis virus (IPNV) by RT-qPCR. The PRV inoculum was prepared by homogenization of the heart, spleen and head kidney. The latter two organs are known to contain higher PRV load than the heart [19].

The shedder fish were injected intraperitoneally with 0.1 mL inoculum, labeled by shortening the outer left maxilla, and placed in a tank containing 50 naïve fish (cohabitants). The samples from challenge # 1 were collected from the cohabitant group; sampling six fish every second week starting at 6 weeks post challenge (wpc) and ending at 14 wpc. In addition, 3 cohabitant fish were sampled at 4 wpc to test for transmission of PRV. At each sampling, peripheral blood from the caudal vein was collected into heparinized vacutainers, and tissue from heart, skeletal muscle, spleen and head kidney were sampled in RNAlater (Life Technologies, Carlsbad, CA, USA) and used for RT-qPCR analysis. Parallel samples from the same organs were harvested in 10% phosphate-buffer formalin, embedded in paraffin, and used for histologic analysis.

At 9 wpc, blood from 15 cohabitant fish was sampled, pooled and centrifuged. After removing the plasma, the blood pellet was diluted 1:4 in phosphate-buffered saline (PBS) and stored at -80 °C. The Ct-value of the diluted blood pellet was confirmed to contain high loads of PRV by RT-qPCR (Ct-value 19.9) and was used as challenge material in Challenge Experiment # 2. It was also confirmed free of other important viral pathogens of salmon, including ISAV, IPNV, SAV and PMCV.

### Challenge experiment # 2

60 shedders and 60 cohabitants with an initial average weight of 90 g were used. The blood pellet prepared in Challenge Experiment # 1 was thawed, diluted 1:3 in PBS, and used as challenge material. Inoculation and labeling of the shedders were performed as in Challenge Experiment # 1. The samples from challenge # 2 were collected from the cohabitant group; six fish were sampled weekly from 2 wpc until 8 wpc. Blood, heart and spleen tissues were sampled as described in Challenge Experiment # 1. Samples for RT-qPCR were taken from heparinized blood as well as the plasma fraction collected after centrifugation at 1000 × *g* for 5 min. The remaining blood was used for isolation of RBC, which were subsequently analyzed by RT-qPCR, flow cytometry, fluorescence and confocal microscopy, and transmission electron microscopy (TEM).

### Isolation of red blood cells

RBC were isolated from the heparinized blood collected from the cohabitant group in Challenge Experiment # 2 by using a previously described Percoll gradient with minor modifications [32]. In short, blood was diluted 1:10 in distilled PBS (dPBS) and a discontinuous Percoll gradient (GE Healthcare, Uppsala, Sweden) was prepared using 1.07 g/mL (49%) and 1.05 g/mL (34%). The diluted blood was layered onto the gradient and centrifuged at 2000 × *g* for 20 min at 4 °C. The RBC were collected from the bottom of the gradient; however a variable amount of erythrocytes was present at the 34%-49% interface. In order to collect the remaining erythrocytes, the interface was re-layered onto a fresh 34%-49% gradient and centrifuged at 500 × *g* for 10 min at 4 °C. The RBC from the bottom of the gradient were collected, pooled with the erythrocytes from the first gradient, and washed twice in PBS. The cells were counted using Countess (Invitrogen, Eugene, Oregon, USA) and resuspended in PBS to a final concentration of 5 × 10^6^ cells/mL. Smears of the isolated RBC stained with Diff-Quik (Dade Behring, Newark, USA) confirmed that the isolated cells were erythrocytes.

### RNA isolation

Total RNA was isolated from 25 mg of tissue stored in RNAlater by homogenization in QIAzol Lysis Reagent (Qiagen, Hilden, Germany) using 5 mm steel beads and TissueLyser II (Qiagen) for 2 × 5 min at 25 Hz. After addition of chloroform and centrifugation, the aqueous phase was collected and mixed with one volume of 70% ethanol before continuing with the RNeasy Mini spin column (Qiagen) as described by the manufacturer. The homogenization and RNA extraction method described above was also used for isolation of total RNA from the heparinized blood samples and isolated RBC using 10 μL blood and 1 × 10^7^ RBC, respectively. For the plasma samples, a combination of Trizol LS (Invitrogen) and RNeasy Mini spin column was used. Briefly, 50 μL plasma was mixed and incubated with Trizol LS before adding chloroform, separating the phases by centrifugation, and then proceeding with the RNeasy Mini spin column as recommended by the manufacturer. The RNA isolated from 50 μL plasma was eluted in 50 μL RNase-free water. RNA was quantified using a NanoDrop ND-1000 spectrophotometer (Thermo Fisher Scientific, Wilmington, DE, USA).

### RT-qPCR

The Qiagen OneStep kit (Qiagen) was used for RT-qPCR. A standard input of 100 ng (5 uL of 20 ng/uL) from the isolated total RNA was used in each reaction in order to compare the amount of PRV in tissue, blood and RBC samples. From the cell free plasma samples 5 μL purified RNA solution, equivalent to 5 μL plasma, was used. The template RNA were denaturated at 95 °C for 5 min prior to RT-qPCR that was performed using the following conditions: 400 nM primer, 300 nM probe, 400 nM dNTPs, 1.26 mM MgCl_2_, 1:100 RNase Out (Invitrogen) and 1 × ROX reference dye with the following cycle parameters: 30 min at 50 °C, 15 min at 94 °C, 40 cycles of 94 °C/15 s, 54 °C/30 s and 72 °C/15 s in a Mx3005P (Stratagene, La Jolla, CA, USA). The samples were run in duplicate, and a sample was defined as positive if both parallel samples had a Ct < 35. The primers and probe used targeted PRV gene segment S1: S1Fwd TGCGTCCTGCGTATGGCACC; S1Rev GGCTGGCATGCCCGAATAGCA; S1probe (FAM) ATCACAACGCCTACCT MGBNFQ.

### Histopathology

Slides from the formalin fixed and paraffin embedded heart material collected from Challenge Experiments # 1 and 2 was stained with hematoxylin-eosin (HE) and evaluated by histopathological examination using conventional light microscopy.

### Flow cytometry

RBC isolated from the cohabitants in Challenge Experiment # 2 were stained using a polyclonal antibody raised in rabbits against putative PRV outer capsid protein ơ1 (Anti-ơ1, #K275) [18], running parallel samples for surface and intracellular labeling. All incubations were performed on ice and the corresponding zero serum (Anti-σ1 Zero #K275) (18) was used as negative control serum. Briefly, cells were plated into 96-well plates at densities of 0.5 × 10^6^ cells per well and washed in staining buffer (PBS + 1% BSA + 0.05% azide). Cells were surface stained with Anti-σ1 (1:10 000) for 30 min and secondary Alexa Fluor 488 conjugated anti-rabbit IgG (Molecular Probes, Eugene, Oregon, USA) (2 mg/mL diluted 1:800) for 30 min using staining buffer in all washes and dilutions. Prior to intracellular staining the cells were fixed and permeabilized by incubation in Cytofix/Cytoperm (Becton Dickinson, San Diego, CA, USA) for 15 min on ice, and the same primary and secondary incubations described above but using Perm/wash (Becton Dickinson) in the dilutions and washing steps. The cells were read on a Gallios Flow Cytometer (Beckman Coulter, Miami, FL, USA) counting 50 000 cells per sample, and the data were analyzed using the Kaluza software (Becton Dickinson). Due to slight variation in the background staining, the flow charts were gated individually, discriminating between negative and positive peaks.

### Immunofluorescence microscopy and confocal microscopy

Samples of the intracellularly stained RBC were prepared for immunofluorescence and confocal microscopy. The nuclei were stained with propidium iodide (0.5 μg/mL, Molecular Probes) and the cells were mounted to glass slides using Fluoroshield (Sigma-Aldrich, St. Louis, USA) and cover slips. Non-saturated images were captured by a Plan-Apochromat 63/1.4 oil objective in a laser scanning confocal microscope (Zeiss Axiovert 200 M fluorescence inverted microscope, equipped with a LSM 510 laser confocal unit 488 nm argon laser and 546 nm helium/neon laser Z). In addition, samples were stained with propidium iodide as described above and the cell suspension was transferred to Countess chamber slides (Invitrogen). Photographs were taken by an inverted fluorescence microscope (Olympus IX81), at 20 × and 40 × magnification.

### Pinacyanol chloride staining of cytoplasmic inclusions

Blood and isolated RBC from cohabitant fish of challenge # 2 were collected 8 wpc and used to prepare blood smears for staining with pinacyanol chloride. The staining was performed as previously described by Leek et al. [26]. Briefly; slides were fixed in methanol for 5 min, and subsequently stained in pinacyanol chloride (prepared by mixing 0.25 g pinacyanol chloride (Santa Cruz Biotechnology) in 35 mL ethanol and 15 mL distilled water) for 1 min and washed with tap water for 5 min. Dry slides were mounted (Aquamount) before microscopy. Pictures were captured at 40 × magnification.

### Transmission electron microscopy (TEM)

RBC isolated from infected fish were fixed overnight at 4 °C in PBS with 1.25% glutaraldehyde and 2% paraformaldehyde, washed twice in PBS, and three times in cacodylate buffer (0.1 M, pH 6.8) The cells were post-fixed with 1% OsO_4_ for 1 h at 4 °C and washed with cacodylate buffer. The cells were then dehydrated through an ethanol series (70, 90, 96 and 100%) and embedded in LR-White resin. Thin sections were cut on an ultra microtome (LEICA EM UC 6). The sections were stained with 4% aqueous uranyl acetate and 1% KMNO_4_ for 10 min, then washed intensively in freshly distilled water. The sections were examined in a FEI MORGAGNI 268, and photographs were recorded using a VELETA camera.

### Data analysis

The Ct-values for PRV in blood were compared with those of the heart, skeletal muscle, spleen and head kidney from the same individual by paired analysis in both Challenge Experiment # 1 and # 2, excluding non-infected individuals. The differences were analysed statistically using Wilcoxon matched pairs signed rank test due to the sample size (*n* = 6). At 6 wpc and 12 wpc in Challenge Experiment # 1 only four and five out of the six sampled fish could be compared respectively. The non-parametric Mann–Whitney test was used when analysing the difference between Ct-values for PRV in blood obtained in Challenge Experiment # 1 and # 2. The side scatter data of the negative and high PRV positive erythrocytes obtained by flow cytometry was analyzed for each time-point by Wilcoxon matched pairs signed rank test due to the non-normality of the data. All statistical analysis described were performed with GraphPad Prism (GraphPad Software inc., USA) and *p*-values of *p* ≤ 0.05 were considered as significant.

## Results

### High PRV load in blood during infection

**Figure 1:**
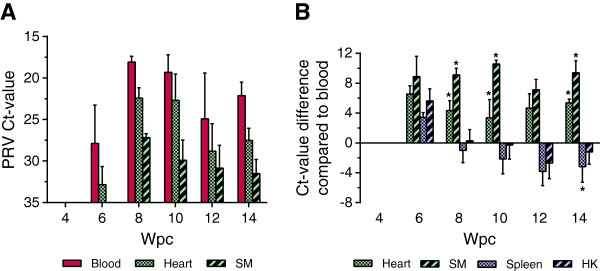
**High PRV load in blood detected in Experimental Challenge # 1. (A)** Detection of PRV by RT-qPCR in Challenge Experiment # 1 presented by mean (and SD) Ct-values in blood, heart and skeletal muscle (SM) (*n* = 6). **(B)** Paired analysis of the PRV Ct-value detected in blood compared with samples from heart, skeletal muscle (SM), spleen, head kidney (HK) of the same fish. The results are presented as the mean Ct-value difference at each time point. Data were analyzed using Wilcoxon matched pairs signed rank test. *p < 0.05.

The RT-qPCR results from Experimental Challenge # 1 revealed a high PRV load in heparinized whole blood samples from the cohabitants. PRV was first detected in blood of cohabitant fish at 6 wpc, and the amount of virus peaked at 8 wpc with a mean Ct-value of 18.6 (± 0.7). High viral loads were present in blood until the end of the study at 14 wpc. The mean Ct-values in blood and those in heart and skeletal muscle, which are the main organs for histopathological changes during HSMI [3], are shown in Figure 1A. The relative amounts of virus in blood were significantly higher than those in both heart and skeletal muscle samples at 8, 10 and 14 wpc (*p* < 0.05) (Figure 1B). Compared to the spleen and head kidney, which are the visceral organs known to contain most virus during an infection [19], the amount of PRV in blood exceeded that of spleen and head kidney in the early phases (6 wpc). The relative PRV load in spleen and head kidney increased compared with that in blood during the infection and in the later phase of infection (14 wpc), there were significantly higher viral loads in the spleen (*p* < 0.05). All Ct-values from Experimental Challenge # 1 are listed in additional file (Additional file 1). Histopathological changes in the heart consistent with an HSMI diagnosis were first observed at 10 wpc in three out of six fish (data not shown).

### Blood cell pellet is a highly effective challenge material

**Figure 2:**
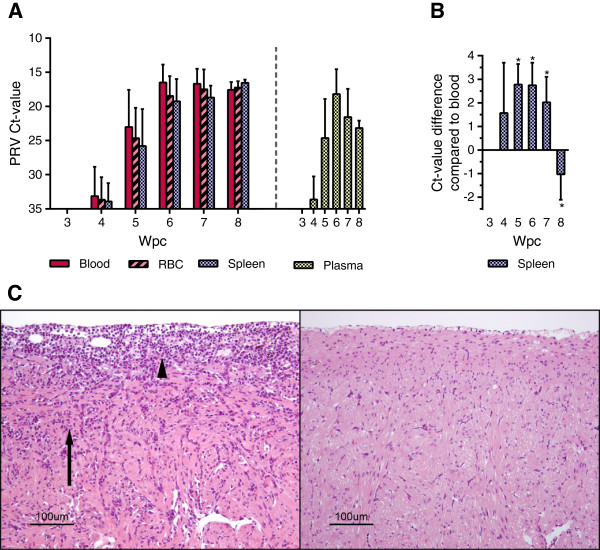
**High PRV load in RBC detected in Experimental Challenge # 2. (A)** Detection of PRV by RT-qPCR in Challenge Experiment # 2 presented by mean (and SD) Ct-values for blood, RBC and spleen. The PCR results from the plasma are shown separately on the right, as the RNA input from the cell free plasma is not directly comparable to the other sample material analyzed. **(B)** Paired analysis of the PRV Ct-value detected in blood compared with spleen from the same fish, presented as mean Ct-value difference at each time point. Data were analyzed using Wilcoxon matched pairs signed rank test. *p < 0.05. **(C)** Histopathological changes in the ventricle of the heart at 7 weeks post challenge (wpc) (left) consistent with HSMI, including epicarditis (arrowhead) and inflammation of the compactum myocardium layer (arrow). For comparison a ventricle without inflammatory changes (sampled at 0 wpc), is shown to the right.

A blood cell pellet prepared from Experimental Challenge # 1 was used as inoculum in shedder fish in Experimental Challenge # 2, and, again, high viral loads were detected in blood in the cohabitants (Figure 2A). PRV was first detected in three out of six fish at 4 wpc (i.e., two weeks earlier than in Experimental Challenge # 1), and at 5 wpc all cohabitant fish sampled were PRV-positive by RT-qPCR. The maximum viral load in blood occurred two weeks earlier than in Experimental Challenge # 1, and plateaued at 6 wpc, with a mean Ct-value of 16.5 (± 0.7). This is significantly higher than the viral loads at the same time point in Experimental Challenge # 1 (*p* < 0.05). The spleen was used as a reference organ for viral load during PRV infection. PRV loads of blood were significantly higher compared with that of the spleen in the early phases of the infection (5–7 wpc) (*p* < 0.05) and shifting to significantly lower levels at 8 wpc (*p* < 0.05) (Figure 2B). Histopathological changes in heart and skeletal muscle consistent with an HSMI diagnosis were first observed at 7 wpc in Experimental Challenge # 2 in four out of six fish (Figure 2C). At 8 wpc, heart lesions were detected in all fish sampled, showing massive epicarditis and infiltration of lymphocytic cells in the compact and spongy myocardium layer of the ventricle.

### High PRV loads in RBC

PRV was first detected in RBC from cohabitants in Experimental Challenge # 2 by RT-qPCR at 4 wpc and plateaued at about 6 to 7 wpc (18.5 ± 2.9 and 17.5 ± 2.9 respectively), thus following the same pattern observed in blood (Figure 2A). Plasma also contained a substantial amount of virus, partly explaining the slightly higher level of virus in whole blood compared with RBC. All Ct-values from Experimental Challenge # 2 are listed in additional file (Additional file 2).

### High numbers of PRV-positive RBC detected by flow cytometry

**Figure 3:**
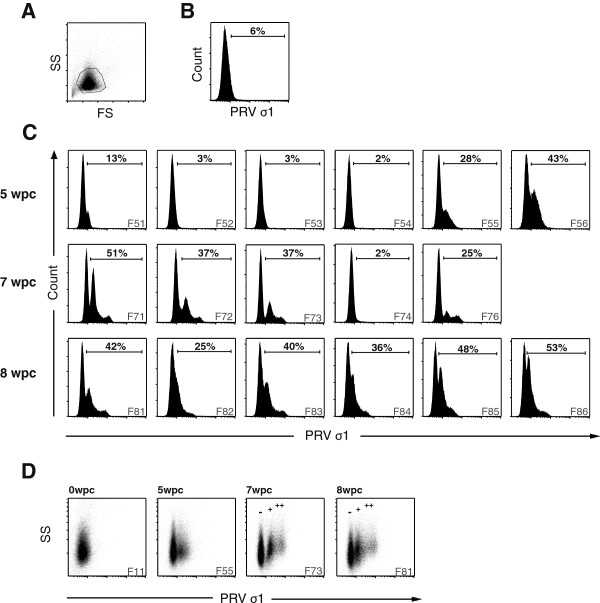
**PRV-positive RBC detected by flow cytometry.** Detection of PRV protein in isolated RBC from Experimental Challenge # 2 by flow cytometry. **(A)** Density plot showing the gating strategy for intracellular staining. FS, forward scatter; SS, side scatter. **(B)** Negative control from 0 weeks post challenge (wpc) for intracellular staining, representing the background fluorescence. **(C)** Flow cytometry result from the intracellular staining at 5, 7 and 8 wpc. 50 000 cells were counted for each sample. Individual F75 was excluded due to technical difficulties. **(D)** Plots of side scatter (SS) and fluorescence intensity (PRV ơ1) from 0, 5, 7 and 8 wpc. PRV negative (-), low positive (+) and high positive (++) population are indicated at 7 and 8 wpc.

The isolated RBC from Experimental Challenge # 2 were gated according to size and granularity to include only intact cells (Figure 3A). Samples from 0 wpc were used as negative controls, providing the background fluorescence signal from PRV-negative samples (Figure 3B). A PRV-positive erythrocyte population was first observed in two out of six individuals (F55, F56) at 5 wpc, as seen by a peak in fluorescence signal for both intracellular and surface staining. The majority of virus protein was detected intracellularly and a large, distinct population of PRV-positive RBC (up to 43% of cells PRV-positive in F56) could be observed (Figure 3C). As shown by RT-qPCR of RBC, the individual fish that were PRV-positive also contained distinctly higher viral loads compared with the rest of the group (Table 1). PRV was also detected on the surfaces of a small number of RBC of the same individuals. The surface staining data from Experimental Challenge # 2 is shown in additional file (Additional file 3).

**Table 1:** Ct-values of PRV in RBC from Challenge # 2

**PRV Ct-value (RBC)**						
**0 wpc**	**3 wpc**	**4 wpc**	**5 wpc**	**6 wpc**	**7 wpc**	**8 wpc**
*F01-F06*	*F31-F36*	*F41-F46*	*F51-F56*	*F61-F66*	*F71-F76*	*F81-F86*
No Ct	No Ct	No Ct	28.2	17.8	16.8	15.7
No Ct	No Ct	No Ct	25.1	23.9	15.5	17.5
No Ct	No Ct	No Ct	29.1	16.8	15.9	16.5
No Ct	No Ct	No Ct	27.1	16.1	23.2	18.1
No Ct	No Ct	No Ct	19.0	16.8	17.5	17.9
No Ct	No Ct	26.9	19.3	19.5	17.9	17.8

RT-qPCR Ct-values of PRV in RBC from individual fish of Experimental Challenge # 2 at 0 and 3–8 weeks post challenge (wpc). Unique identifiers for the six individual fish sampled at each time point are included (Ex. F01-F06 at 0 wpc) counting from the top down of each column.

At 7 wpc, PRV-positive erythrocytes from Experimental Challenge # 2 were observed in four out of five individual fish and a considerable proportion of isolated RBC (25 - 51%) were PRV-positive (Figure 3C). The majority of the virus was found intracellularly. In contrast with the 5 wpc samples, the PRV-positive cells at 7 wpc could be divided into two separate populations, showing low PRV-positive and high PRV-positive staining. The low PRV-positive population was comparable in intensity to the single PRV population detected at 5 wpc, indicating a development of cells containing high PRV levels over time (Figure 3C). At 8 wpc, high numbers of PRV-positive RBC were detected by intracellular staining in all six individuals. However, at 8 WPC the cells no longer clearly formed two populations of low and high PRV-positive staining.

The results from the flow cytometry analysis correlated with the RT-qPCR results, as individual fish with Ct-values below 20 were consistently detected as positive by flow cytometry (Figure 3C, Table 1). Samples with Ct-values above 20 appeared to be below the sensitivity threshold of the flow cytometry analysis, exemplified by F74 at 7 wpc (Figure 3C). At 4 wpc all the RBC samples had Ct-values above 20, producing only a background signal by flow cytometry; at 8 wpc, all samples had Ct-values below 20 and were positive by flow cytometry (Figure 3C).

Throughout the study PRV-positive cells with high fluorescence intensity tended to show an increased side scatter in flow cytometry (Figure 3D). The negative population and high PRV-positive populations were individually gated in each fish, and their mean side scatter was compared using Wilcoxon rank-sum test. The high PRV-positive populations at 7 wpc and 8 wpc had significantly more cells demonstrating higher side scatter properties compared with the negative population (*p* < 0.05) indicating that highly PRV-positive RBC are more granular.

### Viral cytoplasmic inclusions detected in RBC

**Figure 4:**
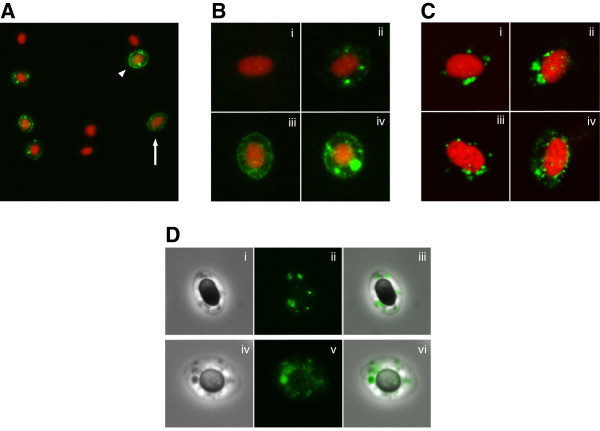
**Immunofluorescence and confocal microscopy of PRV positive RBC.** Fluorescent labeling of the PRV ơ1-protein in erythrocytes (green). Cell nucleus stained with propidium iodide (red). **(A)** Positive RBC detected by immunofluorescence (IF) microscopy. Labeled cells varied in morphology from elongated mature RBC (arrow) to more spherical cells resembling immature erythrocytes (arrowhead). **(B)** IF microscopy of a negative cell (i) and different staining pattern in the cytoplasm including a few smaller inclusions (ii), scattered granular staining (iii), and large cytoplasmic inclusions (iv). **(C)** Confocal microscopy images showing viral inclusions in the perinuclear region (i-iii) and a more scattered staining pattern (iv). **(D)** Cytoplasmic inclusion detected by phase contrast (i, iv) and IF (ii, v) microscopy, and co-localized by image overlay (iii, vi).

PRV-positive erythrocytes were identified by immunofluorescence microscopy by granular staining in the cytoplasm (Figure 4A). The staining was observed as inclusions that varied in size and number from a few large inclusions to multiple smaller inclusions, or diffuse granular staining throughout the cytoplasm (Figure 4B). The morphology of the positive RBC varied from typical mature erythrocytes, with an ellipsoidal shape, to more circular cells. The PRV-positive staining was confirmed to be cytoplasmic by confocal microscopy, and the viral inclusions were primarily detected in the perinuculear region, but also found scattered in the cytoplasm (Figure 4C). Small inclusions were also observed in the nucleus in some cells (Figure 4C, ii-iv). Cytoplasmic inclusions were also observed in some RBC by phase contrast microscopy, and these inclusions co-localized with the immunofluorescent staining of PRV (Figure 4D).

### Viral inclusions detected by pinacyanol staining

**Figure 5:**
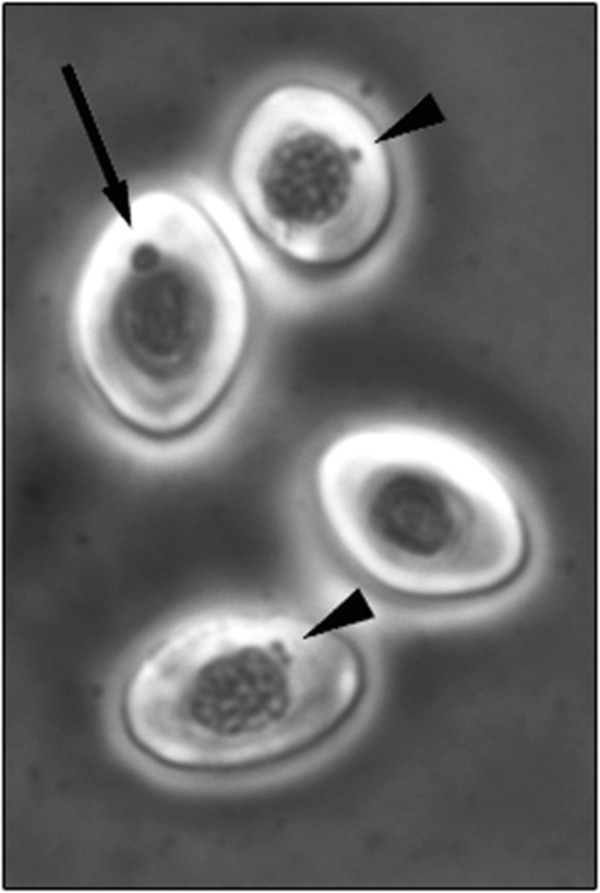
**Pinacyanol chloride staining of RBC.** Cytoplasmic inclusions detected in blood smears from PRV positive fish. Both small (arrowhead) and large inclusions (arrow) were observed in cytosol of RBC. Picture were taken at 40 × magnification.

Cytoplasmatic inclusions were detected using pinacyanol chloride staining method observed as single or several small, or single big inclusion(s) were observed (Figure 5). Slides stained with pinacyanol chloride were best observed without mounting media as the stain tended to leak into the mounting media.

### Reovirus-like particles observed by TEM

**Figure 6:**
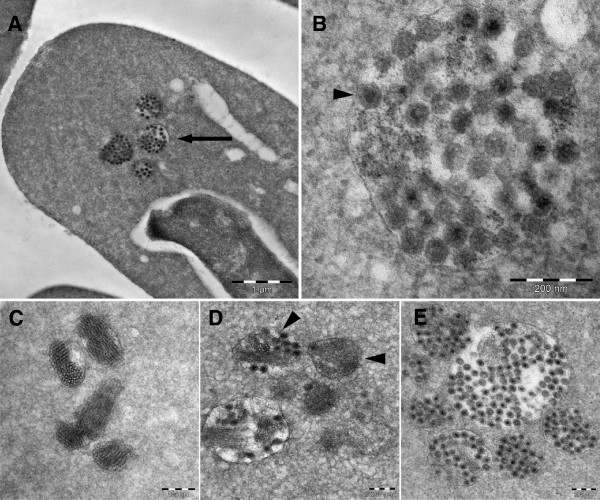
**Transmission electron micrographs of infected RBC. A)** Erythrocyte with viral inclusions (arrow). **B)** Inclusions containing reovirus-like particles. **C)** Inclusions with lamellar structure. **D)** Inclusions with a mixture of viral particles and lamellar structures (arrowhead). Some of the inclusions are surrounded by an intact, or partially intact membrane-like structures. **E)** Inclusions containing numerous reovirus-like particles.

The TEM analysis of RBC from infected fish revealed cytoplasmic inclusions (Figure 6A), which contained reovirus-like particles (Figure 6B). The inclusions varied in content and size from approximately 100–1000 nm. Some of the inclusions consisted only of lamellar structures up to 500 nm (Figure 6C). The larger inclusions contained both these lamellar structures and reovirus-like particles (Figure 6D), whereas the biggest inclusions contained only viral particles (Figure 6E). These differences may represent the various stages of the viral replication cycle. Many of inclusions were enclosed within a membrane-like structure that seemed to be partly or fully intact (Figures 6B-E). The viral particles were naked with an outer diameter of approximately 72 nm (Figures 6B and E). A more electron dense core, with a diameter of about 30 nm, was visible in many of the particles. These features are consistent with descriptions of other members of the reovirus family.

## Discussion

In this study we demonstrate that RBC are major target cells for PRV. RBC contained high levels of PRV protein and RNA as shown by flow cytometry and RT-qPCR respectively. PRV protein was located to cytoplasmic inclusions resembling viral factories as observed by immunofluorescence and confocal microscopy. Finally, these inclusions were shown by TEM to contain reovirus-like particles. All together, these observations show PRV infection of Atlantic salmon erythrocytes.

In Experimental Challenge # 1, we found that a major part of the PRV load is present in blood and the PRV levels in blood exceeded those of the organs displaying pathological changes i.e. heart and skeletal muscle. Based on these initial results, a blood cell pellet was used as inoculum in Experimental Challenge # 2, and this generated high loads of PRV in cohabitant fish. Currently there are no in vitro assays to quantify infectious units of PRV. However, the successful transmission of PRV to cohabitant fish after injection of blood cell pellet to the shedder fish, confirmed the presence of infectious virus in blood. The results from RT-qPCR and flow cytometry analysis of Challenge Experimental Challenge # 2 revealed that the blood borne PRV were mainly present in the erythrocyte fraction.

Immunofluorescence and confocal microscopy demonstrated that PRV condensed into globular structures, localized to the cytoplasm of infected RBC. Furthermore, the flow cytometry analysis showed that highly PRV-positive RBC had a significantly higher side scatter, and were thus more granular than the PRV-negative RBC in an infected individual. Orthoreovirus-infected cells are associated with phase-dense inclusions in the cytoplasm, known as viral factories [33,34]. These viral factories contain large amounts of viral proteins [34,35], dsRNA [36], and partially or fully assembled particles [34], and are considered to be the location where viral RNA replication and packaging, and assembly of progeny particles occurs [33,37]. The inclusions observed in this study appear very similar to those described for orthoreoviruses; more specifically, they resemble the globular viral factories seen in MRV type 3 prototype strain Dearing (T3D) infected cells, rather than the microtubule-associated filamentous factories of the type 1 prototype strain Lang (T1L) [38,39]. The capacity of MRV to form viral factories facilitates successful viral replication [40-44].

The viral inclusions observed in this study were found by TEM to contain large amounts of reovirus-like particles. The features were similar to those reported for the *Reoviridae* family, with naked virions with an outer diameter of 72 nm and an electron-dense center of 30 nm. Negative- stain EM of MRV has indicated a virion diameter of 73 nm, while the infectious subviral particles (ISVPs) and cores, that are two types of partially uncoated MRV particles, were 64 and 51 nm respectively [45]. The diameters obtained by cryo-EM, in which reduce shrinking artifacts, are 85, 80 and 60 nm for virions, ISVPs and cores respectively [46,47]. If the electron-dense center reported in this study represents the reovirus core, then it is smaller than for MRV. It could, however, represent the centrally condensed RNA genome [35,48,49]. Further studies including gradient ultracentrifugation and negative staining are needed to obtain a more detailed description of the PRV particle.

The inclusions varied in size from 100 to 1000 nm and were often located in the perinuclear region. The content of the inclusions differed, with some filled with reovirus-like particles while others contained a mixture of virus particles and a more homogenous material with lamellar structures. The nature of these lamellar structures was not determined. MRV inclusions have been shown to contain both microtubules and intermediate filaments [35,50,51], and Sharpe et al. have described the intermediate filament, vimentin, being rearranged in reovirus-infected CV-1 cells, both surrounding and incorporating into the viral inclusions [52]. Most of the inclusions detected in our study were encapsulated by a membrane-like structure, whereas in others the membrane seemed to be broken, incomplete, or absent. MRV inclusions are not associated with membranes or other cellular organelles [34,53,54]. Whether the membrane-like structures observed in our study were lipids or proteinaceous in nature was not determined.

There have been a number of reports of viral inclusions in salmonid erythrocytes that have been collectively termed EIBS [26]. No specific viruses have been recognized as causative agents and erythrocytic inclusions assumed to be of viral origin have been observed in salmonid species in both the Atlantic and Pacific, including Atlantic salmon, rainbow trout (*O. mykiss*), Chinook salmon (*O. tshawythscha*) and Coho salmon (*O. kisutch*) [21,23,55]. The PRV particles and inclusions described in our study have a striking resemblance to several of the previously reported viral inclusions described as EIBS [21-25,55]. Pinacyanol chloride staining has previously been used in identification of EIBS [26,56], and in the present study we observed stained inclusions using this techniques. These earlier reports all describe membrane-bound viral inclusions containing a naked virus of the same size as that of the current study and have been observed in both healthy and diseased fish, the latter with clinical signs including anemia [25,56]. A lamellar structure, as described in our study, was also noted in many of these reports [22,24,25]. Our findings demonstrate that erythrocytes are a major target cell for PRV in Atlantic salmon that can be observed as erythrocytic inclusions. Furthermore, it indicates that PRV is probably associated with at least some of the EIBS previously reported. However, the term EIBS comprise a number of reports from several different salmonid species. The results from the present study should prompt further investigation into the presence of PRV in EIBS infected specimens from both Atlantic and Pacific salmonid species.

Recent studies have concluded that PRV is closely related to the *Orthoreovirus* genus and that it encodes a protein homologous to the MRV ơ1 cell attachment protein [5,6]*.* MRV agglutinate erythrocytes via the ơ1 protein, a binding mediated by a sialic acid binding motif [27,57] that is partly conserved in PRV σ1 [6]. Given the phylogenetic relationship between MRV and PRV, it is interesting to note that virus and erythrocyte interaction is conserved. PRV could serve as an interesting model for investigating phylogenetic relationships in the family *Reoviridae* as it is currently the only known fish virus related to the *Orthoreovirus* genus.

The major function of mammalian erythrocytes is oxygen transport, and mammalian RBC undergo enucleation and loss of organelles before entering circulation [58]. This evolutionary trait benefits their oxygen-carrying capacity and has also made them more resistant to viral infections. Cold-water fish, like Atlantic salmon, have a lower oxygen demand and slower metabolism, and are therefore less dependent on high oxygen transport. An extreme example of low oxygen demand is Antarctic icefish (Channichthyidae) that lack both hemoglobin and erythrocytes [59,60]. Most non-mammalian erythrocytes, including those of fish, are nucleated and contain organelles, and can therefore potentially serve as hosts for viral replication [61]. In this study, more than 50% of the RBC of some individual fish were PRV-infected, as demonstrated by flow cytometry. As the sensitivity of the flow cytometry analysis enabled detection of infected RBC only in those individual fish with RBC RT-qPCR Ct-values of below 20, the prevalence of PRV-infected RBC is probably higher. Considering that RBC are the most abundant cells in blood, by extrapolation, total viral load in an infected fish is substantial.

Although mature erythrocytes in humans cannot support productive viral infections, Colorado tick fever virus (CTFV), a reovirus of the genus *Coltivirus*, has been detected in mature erythrocytes [62,63]. This is thought to be the result of infection of nucleated immature cells rather than of direct entry and replication of CTFV in mature erythrocytes, as CTFV has been shown to replicate in erythroblasts and reticulocytes of infected mice and in human hematopoietic progenitor cells [64,65]. Among the PRV-positive erythrocytes detected by immunofluorescence microscopy variations in cell morphology were observed, with some identified as typical ellipsoidal mature erythrocytes whereas others resembled immature erythrocytes with a more circular cellular shape [66]. In Atlantic salmon farming, factors like reduced oxygen supply and acute anemia stimulate erythropoiesis and would therefore be expected to increase the relative proportion of juvenile cells [67,68]. However, both young and old RBC from rainbow trout have been shown to respond to stress by altered protein production [31]. The spherical RBC observed in this study could be due to stress-induced swelling of RBC [69], and not necessarily indicate immature erythrocytes. Future studies should investigate the replicative potential for PRV in hematopoietic cells.

Several studies have been conducted in order to investigate the significance of PRV infection on the health of farmed Atlantic salmon. PRV has been shown to be the etiological cause of HSMI, as the cardiac viral load correlates with disease development and PRV antigens have been detected in cardiomyocytes, coinciding with the course of the disease [2,18]. However, our study shows that infection of red blood cells is an important feature of PRV infection that is not encompassed by the diagnosis of HSMI. Although HSMI is caused by PRV, the disease should probably be considered as only one possible outcome of a PRV infection. PRV infection of erythrocytes could have broader implications for fish health, irrespective of the presence of heart lesions.

Lesions in the spleen and head kidney are not included in the diagnostic criteria of HSMI. However, these blood-filled organs contain more virus compared to heart and skeletal muscle [19], which was confirmed in this study. A proportion of these high viral loads are likely due to PRV-infected RBC. In addition, both organs are immunologically important for clearance of viral infected circulating cells. Nevertheless, PRV infection in non-RBC cells in the head kidney or spleen should not be excluded. It is also worth noting that head kidney and spleen are the main hematopoietic organs in Atlantic salmon. PRV positive cells resembling leukocytes have been observed by immunohistochemistry on heart sections from HSMI diseased fish, but these cells have not been further characterized [18]. In our study it cannot be excluded that a few leukocytes could have been co-purified along with RBC. However, the number would be very low compared to the massive numbers of infected RBC found in flow cytometry. Double staining using PRV antibody and specific markers for populations of Atlantic salmon leukocytes could be attempted in future studies to identify potential non-RBC target cells.

PRV is ubiquitous in healthy farmed Atlantic salmon, and occurs at lower prevalences in wild Atlantic salmon and brown trout (*Salmo trutta* L.) in Norway, and wild chum salmon (*Oncorhynchus keta*), rainbow trout (*O. mykiss*) and cutthrout trout (*O. clarkii*) in Canada [7,10]. The widespread occurrence of PRV in healthy farmed and wild fish could reflect infection in erythrocytes at levels that do not lead to the heart and skeletal muscle lesions seen in HSMI outbreaks. Field observations indicate that PRV is present in farmed Atlantic salmon long after cessation of an HSMI outbreak [8]. Persistent infection of erythrocytes could explain these findings, as fish erythrocytes are thought to circulate for up to 6 months [31]. It is important to note that the presence of PRV in circulating erythrocytes will influence the amount of virus detected by qPCR screening of any organ, including heart, skeletal muscle, head kidney and spleen. Residual blood containing PRV-positive cells would affect the qPCR results obtained, even if resident cells are not infected.

In conclusion, demonstration of PRV infection of erythrocytes provides new information about HSMI pathogenesis, and establishes PRV as an important factor of viral erythrocytic inclusions.

## Abbreviations

ARV: Avian orthoreovirus; dsRNA: Double-stranded RNA; EM: Electron microscopy; EIBS: Erythrocytic inclusion body syndrome; HE: Hematoxylin-eosin; HSMI: Heart and skeletal muscle inflammation; IP: Intraperitoneally; IPNV: Infectious pancreatic necrosis virus; ISAV: Infectious salmon anemia virus; MRV: Mammalian orthoreovirus; PMCV: Piscine myocarditis virus; PBS: Phosphate-buffered saline; PRV: Piscine orthoreovirus; RBC: Red blood cells; SAV: Salmonid alfavirus; TEM: Transmission electron microscopy; VLP: Virus like particle; WPC: Weeks post challenge.

## Competing interests

The authors declare that they have no competing interests.

## Authors’ contributions

ØWF participated in the overall design of the study, performed the RBC isolation, flow cytometry, immunofluorescence microscopy and electron microscopy, participated in RNA isolation and RT-qPCR, helped in interpretation of the overall data and drafted the manuscript. MKD participated in the overall design of the study, performed the RNA isolation and RT-qPCR, helped in interpretation of the overall data and drafting the manuscript. THL participated in isolation of RBC and flow cytometry, helped in interpretation of these data and revised the manuscript. IBN participated in RNA isolation and RT-qPCR, helped in the interpretation of these data and revised the manuscript. ML designed and carried out the challenge experiments and revised the manuscript. CW designed and carried out the challenge experiments and revised the manuscript. CMO carried out the confocal imaging and pinacyanol staining, helped in the interpretation of these data and revised the manuscript. AKS helped in the development the flow cytometry assay, interpretation of the data and revised the manuscript. ER participated in the overall design and coordination of the study, helped in interpretation of the overall data and drafting the manuscript. All authors read and approved the final manuscript.

## Supplementary Material

Additional file 1 **RT-qPCR Ct-values of PRV from Challenge Experiment # 1.** RTq-PCR Ct-values of PRV in blood, heart, skeletal muscle (SM), spleen and head kidney (HK) from Challenge Experiment # 1 sampled at 0, 4, 6, 8, 10, 12 and 14 weeks post challenge (wpc). Unique identifiers for the individual fish sampled are included (Ex. F101-F106). The mean Ct-value and standard deviation (Mean (± SD)) for the different tissues are shown in the righthand column. Samples with no Ct-value were assigned the value 35.0 when used in calculation of the mean. Na: Not available. Click here for file

Additional file 2 **RT-qPCR Ct-values of PRV from Challenge Experiment # 2.** RTq-PCR Ct-values of PRV in blood, RBC, spleen and plasma from Challenge Experiment # 2 sampled at 0 and 3–8 weeks post challenge (wpc). Unique identifiers for the individual fish sampled are included (Ex. F01-F06). The mean Ct-value and standard deviation (Mean (± SD)) for the different material are shown in the right-hand column. Samples with no Ct-value were assigned the value 35.0 when used in calculation of the mean. Click here for file

Additional file 3 **Surface detection of PRV protein in isolated RBC from Challenge Experiment # 2 by flowcytometry.(A)** Density plot showing the gating strategy for surface staining. FS, forward scatter; SS, side scatter. **(B)** Negative control from 0 weeks post challenge (wpc) for surface staining representing the background fluorescence. **(C)** Flow cytometry results from the surface staining at 5, 7 and 8 wpc. 50 000 cells were counted for each sample. Individual F75 was excluded due to technical difficulties. Click here for file

## References

[B1] Kongtorp RT Kjerstad A Taksdal T Guttvik A Falk K Heart and skeletal muscle inflammation in Atlantic salmon, *Salmo salar* L.: a new infectious disease J Fish Dis 2004 27 351 358 10.1111/j.1365-2761.2004.00549.x 15189375

[B2] Palacios G Løvoll M Tengs T Hornig M Hutchison S Hui J Kongtorp RT Savji N Bussetti AV Solovyov A Kristoffersen AB Celone C Street C Trifonov V Hirschberg DL Rabadan R Egholm M Rimstad E Lipkin WI Heart and skeletal muscle inflammation of farmed salmon is associated with infection with a novel reovirus PLoS One 2010 5 e11487 10.1371/journal.pone.0011487 20634888 PMC2901333

[B3] Kongtorp RT Taksdal T Lyngøy A Pathology of heart and skeletal muscle inflammation (HSMI) in farmed Atlantic salmon *Salmo salar* Dis Aquat Organ 2004 59 217 224 15264718 10.3354/dao059217

[B4] Mikalsen AB Haugland Ø Rode M Solbakk IT Evensen Ø Atlantic salmon reovirus infection causes a CD8 T cell myocarditis in Atlantic salmon (*Salmo salar* L.) PLoS One 2012 7 e37269 10.1371/journal.pone.0037269 22693625 PMC3367920

[B5] Key T Read J Nibert ML Duncan R Piscine reovirus encodes a cytotoxic, non-fusogenic, integral membrane protein and previously unrecognized virion outer-capsid proteins J Gen Virol 2013 94 1039 1050 10.1099/vir.0.048637-0 23343626

[B6] Markussen T Dahle MK Tengs T Løvoll M Finstad ØW Wiik-Nielsen CR Grove S Lauksund S Robertsen B Rimstad E Sequence analysis of the genome of piscine orthoreovirus (PRV) associated with heart and skeletal muscle inflammation (HSMI) in Atlantic salmon (*Salmo salar*) PLoS One 2013 8 e70075 10.1371/journal.pone.0070075 23922911 PMC3726481

[B7] Kibenge MJ Iwamoto T Wang Y Morton A Godoy MG Kibenge FS Whole-genome analysis of piscine reovirus (PRV) shows PRV represents a new genus in family Reoviridae and its genome segment S1 sequences group it into two separate sub-genotypes Virol J 2013 10 230 10.1186/1743-422X-10-230 23844948 PMC3711887

[B8] Løvoll M Alarcon M Bang JB Taksdal T Kristoffersen AB Tengs T Quantification of piscine reovirus (PRV) at different stages of Atlantic salmon *Salmo salar* production Dis Aquat Organ 2012 99 7 12 10.3354/dao02451 22585298

[B9] Wiik-Nielsen CR Ski P-MR Aunsmo A Løvoll M Prevalence of viral RNA from piscine reovirus and piscine myocarditis virus in Atlantic salmon, *Salmo salar* L., broodfish and progeny J Fish Dis 2012 35 169 171 10.1111/j.1365-2761.2011.01328.x 22175824

[B10] Garseth AH Fritsvold C Opheim M Skjerve E Biering E Piscine reovirus (PRV) in wild Atlantic salmon, *Salmo salar* L., and sea-trout, *Salmo trutta* L., in Norway J Fish Dis 2013 36 483 493 10.1111/j.1365-2761.2012.01450.x 23167652

[B11] Walters MN Joske RA Leak PJ Stanley NF Murine infection with reovirus: I. Pathology of the acute phase Br J Exp Pathol 1963 44 427 436 14079016 PMC2095250

[B12] Attoui H Mertens PPC Becnel J Belaganahalli S Bergoin M Brussaard CP Chappell JD Ciarlet M del Vas M Dermody TS Dormitzer PR Duncan R Fcang Q Graham R Guglielmi KM Harding RM Hillman B Makkay A Marzachì C Matthijnssens J Milne RG Mohd Jaafar F Mori H Noordeloos AA Omura T Patton JT Rao S Maan M Stoltz D Suzuki N King AMQ, Adams MJ, Carstens EB, Lefkowitz EJ Reoviridae Virus Taxonomy: Ninth Report of the International Committee on Taxonomy of Viruses 2012 Amsterdam: Elsevier/Academic Press 541 637

[B13] Ni Y Kemp MC A comparative study of avian reovirus pathogenicity: virus spread and replication and induction of lesions Avian Dis 1995 39 554 566 10.2307/1591809 8561741

[B14] Shivaprasad HL Franca M Woolcock PR Nordhausen R Day JM Pantin-Jackwood M Myocarditis associated with reovirus in turkey poults Avian Dis 2009 53 523 532 10.1637/8916-050309-Reg.1 20095152

[B15] Johansen R (ed.) The health situation in norwegian aquaculture 2012 2013 Oslo: Norwegian Veterinary Institute

[B16] Ferguson HW Kongtorp RT Taksdal T Graham D Falk K An outbreak of disease resembling heart and skeletal muscle inflammation in Scottish farmed salmon, Salmo salar L., with observations on myocardial regeneration J Fish Dis 2005 28 119 123 10.1111/j.1365-2761.2004.00602.x 15705157

[B17] Kongtorp RT Halse M Taksdal T Falk K Longitudinal study of a natural outbreak of heart and skeletal muscle inflammation in Atlantic salmon, *Salmo salar* L J Fish Dis 2006 29 233 244 10.1111/j.1365-2761.2006.00710.x 16635063

[B18] Finstad ØW Falk K Løvoll M Evensen Ø Rimstad E Immunohistochemical detection of piscine reovirus (PRV) in hearts of Atlantic salmon coincide with the course of heart and skeletal muscle inflammation (HSMI) Vet Res 2012 43 27 10.1186/1297-9716-43-27 22486941 PMC3384478

[B19] Løvoll M Wiik-Nielsen J Grove S Wiik-Nielsen CR Kristoffersen AB Faller R Poppe T Jung J Pedamallu CS Nederbragt AJ Meyerson M Rimstad E Tengs T A novel totivirus and piscine reovirus (PRV) in Atlantic salmon (*Salmo salar*) with cardiomyopathy syndrome (CMS) Virol J 2010 7 309 10.1186/1743-422X-7-309 21067578 PMC2994541

[B20] Kongtorp RT Taksdal T Studies with experimental transmission of heart and skeletal muscle inflammation in Atlantic salmon, *Salmo salar* L J Fish Dis 2009 32 253 262 10.1111/j.1365-2761.2008.00983.x 19236557

[B21] Watanabe K Karlsen M Devold M Isdal E Litlabo A Nylund A Virus-like particles associated with heart and skeletal muscle inflammation (HSMI) Dis Aquat Organ 2006 70 183 192 16903229 10.3354/dao070183

[B22] Michak P Smith CE Hopper K Erythrocytic inclusion body syndrome: a light and electron microscopic study of infected erythrocytes of chinook *Oncorhynchus tshawytscha* and coho *0. kisutch* salmon Dis Aquat Organ 1992 12 229 233

[B23] Rodger HD Erythrocytic inclusion body syndrome virus in wild Atlantic salmon, Salmo salar L J Fish Dis 2007 30 411 418 10.1111/j.1365-2761.2007.00831.x 17584438

[B24] Graham DA Curran W Rowley HM Cox DI Cockerill D Campbell S Todd D Observation of virus particles in the spleen, kidney, gills and erythrocytes of Atlantic salmon, *Salmo salar* L., during a disease outbreak with high mortality J Fish Dis 2002 25 227 234 10.1046/j.1365-2761.2002.00358.x

[B25] Lunder T Thorud K Holt RA Rohovec JS Particles similar to the virus of erythrocytic inclusion body syndrome, EIBS, detected in Atlantic salmon (*Salmo salar*) in Norway Bull Eur Assoc Fish Pathol 1990 10 21 23

[B26] Leek SL Viral erythrocytic inclusion body syndrome (EIBS) occurring in juvenile spring chinook salmon (*Oncorhynchus tshawytscha*) reared in freshwater Can J Fish Aquat Sci 1987 44 685 688 10.1139/f87-083

[B27] Reiter DM Frierson JM Halvorson EE Kobayashi T Dermody TS Stehle T Crystal structure of reovirus attachment protein ơ1 in complex with sialylated oligosaccharides PLoS Pathog 2011 7 e1002166 10.1371/journal.ppat.1002166 21829363 PMC3150272

[B28] Chappell JD Gunn VL Wetzel JD Baer GS Dermody TS Mutations in type 3 reovirus that determine binding to sialic acid are contained in the fibrous tail domain of viral attachment protein sigma1 J Virol 1997 71 1834 1841 9032313 10.1128/jvi.71.3.1834-1841.1997 PMC191253

[B29] Rosen L Serologic grouping of reoviruses by hemagglutination-inhibition Am J Hyg 1960 71 242 249 14438891 10.1093/oxfordjournals.aje.a120107

[B30] Dermody TS Nibert ML Bassel-Duby R Fields BN A sigma 1 region important for hemagglutination by serotype 3 reovirus strains J Virol 1990 64 5173 5176 2398540 10.1128/jvi.64.10.5173-5176.1990 PMC248012

[B31] Lund SG Phillips MC Moyes CD Tufts BL The effects of cell ageing on protein synthesis in rainbow trout (*Oncorhynchus mykiss*) red blood cells J Exp Biol 2000 203 2219 2228 10862734 10.1242/jeb.203.14.2219

[B32] Bridle A Nosworthy E Polinski M Nowak B Evidence of an antimicrobial-immunomodulatory role of Atlantic salmon cathelicidins during infection with Yersinia ruckeri PLoS One 2011 6 e23417 10.1371/journal.pone.0023417 21858109 PMC3153500

[B33] Fields BN Raine CS Baum SG Temperature-sensitive mutants of reovirus type 3: defects in viral maturation as studied by immunofluorescence and electron microscopy Virology 1971 43 569 578 10.1016/0042-6822(71)90282-0 4107549

[B34] Rhim JS Jordan LE Mayor HD Cytochemical, fluorescent-antibody and electron microscopic studies on the growth of reovirus (ECHO 10) in tissue culture Virology 1962 17 342 355 10.1016/0042-6822(62)90125-3 14491769

[B35] Dales S Gomatos PJ Hsu KC The uptake and development of reovirus in strain L cells followed with labeled viral ribonucleic acid and ferritin-antibody conjugates Virology 1965 25 193 211 10.1016/0042-6822(65)90199-6 14297208

[B36] Silverstein SC Schur PH Immunofluorescent localization of double-stranded RNA in reovirus-infected cells Virology 1970 41 564 566 10.1016/0042-6822(70)90178-9 4912824

[B37] Spendlove RS Lennette EH Knight CO Chin JN Development of viral antigen and infectious virus in HeLa cells infected with reovirus J Immunol 1963 90 548 553 14082016

[B38] Broering TJ Parker JS Joyce PL Kim J Nibert ML Mammalian reovirus nonstructural protein μNS forms large inclusions and colocalizes with reovirus microtubule-associated protein μ2 in transfected cells J Virol 2002 76 8285 8297 10.1128/JVI.76.16.8285-8297.2002 12134034 PMC155143

[B39] Parker JS Broering TJ Kim J Higgins DE Nibert ML Reovirus core protein μ2 determines the filamentous morphology of viral inclusion bodies by interacting with and stabilizing microtubules J Virol 2002 76 4483 4496 10.1128/JVI.76.9.4483-4496.2002 11932414 PMC155082

[B40] Arnold MM Murray KE Nibert ML Formation of the factory matrix is an important, though not a sufficient function of nonstructural protein μNS during reovirus infection Virology 2008 375 412 423 10.1016/j.virol.2008.02.024 18374384 PMC2486453

[B41] Becker MM Goral MI Hazelton PR Baer GS Rodgers SE Brown EG Coombs KM Dermody TS Reovirus ơNS protein is required for nucleation of viral assembly complexes and formation of viral inclusions J Virol 2001 75 1459 1475 10.1128/JVI.75.3.1459-1475.2001 11152519 PMC114052

[B42] Kobayashi T Chappell JD Danthi P Dermody TS Gene-specific inhibition of reovirus replication by RNA interference J Virol 2006 80 9053 9063 10.1128/JVI.00276-06 16940517 PMC1563907

[B43] Kobayashi T Ooms LS Chappell JD Dermody TS Identification of functional domains in reovirus replication proteins μNS and μ2 J Virol 2009 83 2892 2906 10.1128/JVI.01495-08 19176625 PMC2655549

[B44] Becker MM Peters TR Dermody TS Reovirus ơNS and μNS proteins form cytoplasmic inclusion structures in the absence of viral infection J Virol 2003 77 5948 5963 10.1128/JVI.77.10.5948-5963.2003 12719587 PMC154006

[B45] Borsa J Copps TP Sargent MD Long DG Chapman JD New intermediate subviral particles in the in vitro uncoating of reovirus virions by chymotrypsin J Virol 1973 11 552 564 4349495 10.1128/jvi.11.4.552-564.1973 PMC355137

[B46] Dryden KA Wang G Yeager M Nibert ML Coombs KM Furlong DB Fields BN Baker TS Early steps in reovirus infection are associated with dramatic changes in supramolecular structure and protein conformation: analysis of virions and subviral particles by cryoelectron microscopy and image reconstruction J Cell Biol 1993 122 1023 1041 10.1083/jcb.122.5.1023 8394844 PMC2119633

[B47] Metcalf P Cyrklaff M Adrian M The three-dimensional structure of reovirus obtained by cryo-electron microscopy EMBO J 1991 10 3129 3136 1915287 10.1002/j.1460-2075.1991.tb04874.x PMC453034

[B48] Luftig RB Kilham SS Hay AJ Zweerink HJ Joklik WK An ultrastructural study of virions and cores of reovirus type 3 Virology 1972 48 170 181 10.1016/0042-6822(72)90124-9 4111673

[B49] Mayor HD Jamison RM Jordan LE Vanmitchell M Reoviruses. II. Structure and composition of the virion J Bacteriol 1965 89 1548 1556 14291595 10.1128/jb.89.6.1548-1556.1965 PMC277691

[B50] Spendlove RS Lennette EH Chin JN Knight CO Effects of antimitotic agents on intracellular reovirus antigen Cancer Res 1964 24 1826 1833 14230929

[B51] Dales S Association between the spindle apparatus and reovirus Proc Natl Acad Sci U S A 1963 50 268 275 10.1073/pnas.50.2.268 14060643 PMC221166

[B52] Sharpe AH Chen LB Fields BN The interaction of mammalian reoviruses with the cytoskeleton of monkey kidney CV-1 cells Virology 1982 120 399 411 10.1016/0042-6822(82)90040-X 7201720

[B53] Mayor HD Studies on reovirus. III. A labile, single-stranded ribonucleic acid associated with the late stages of infection J Natl Cancer Inst 1965 35 919 925 5323147

[B54] Gomatos PJ Tamm I Dales S Franklin RM Reovirus type 3: physical characteristics and interaction with L cells Virology 1962 17 441 454 10.1016/0042-6822(62)90139-3 13900037

[B55] Meyers TR First report of erythrocytic inclusion body syndrome (EIBS) in chinook salmon *Oncorhynchus tshawytscha* in Alaska, USA Dis Aquat Organ 2007 76 169 172 17760390 10.3354/dao076169

[B56] Piacentini SC Rohovec JS Fryer JL Epizootiology of erythrocytic inclusion body syndrome J Aquat Anim Health 1989 1 173 179 10.1577/1548-8667(1989)001<0173:EOEIBS>2.3.CO;2

[B57] Chappell JD Duong JL Wright BW Dermody TS Identification of carbohydrate-binding domains in the attachment proteins of type 1 and type 3 reoviruses J Virol 2000 74 8472 8479 10.1128/JVI.74.18.8472-8479.2000 10954547 PMC116358

[B58] Parmley RT Rowley HM, Ratcliffe NA Mammals Vertebrate Blood Cells 1988 Cambridge: Cambridge University Press 337 424

[B59] Brett JR The metabolic demand for oxygen in fish, particularly salmonids, and a comparison with other vertebrates Respir Physiol 1972 14 161 170 10.1016/0034-5687(72)90025-4 5042150

[B60] Garofalo F Pellegrino D Amelio D Tota B The Antarctic hemoglobinless icefish, fifty five years later: a unique cardiocirculatory interplay of disaptation and phenotypic plasticity Comp Biochem Physiol A Mol Integr Physiol 2009 154 10 28 10.1016/j.cbpa.2009.04.621 19401238

[B61] Claver JA Quaglia AIE Comparative morphology, development, and function of blood cells in nonmammalian vertebrates J Exot Pet Med 2009 18 87 97 10.1053/j.jepm.2009.04.006

[B62] Emmons RW Oshiro LS Johnson HN Lennette EH Intra-erythrocytic location of Colorado tick fever virus J Gen Virol 1972 17 185 195 10.1099/0022-1317-17-2-185 4564691

[B63] Hughes LE Casper EA Clifford CM Persistence of Colorado tick fever virus in red blood cells Am J Trop Med Hyg 1974 23 530 532 4823801 10.4269/ajtmh.1974.23.530

[B64] Philipp CS Callaway C Chu MC Huang GH Monath TP Trent D Evatt BL Replication of Colorado tick fever virus within human hematopoietic progenitor cells J Virol 1993 67 2389 2395 8445735 10.1128/jvi.67.4.2389-2395.1993 PMC240408

[B65] Oshiro LS Dondero DV Emmons RW Lennette EH The development of Colorado tick fever virus within cells of the haemopoietic system J Gen Virol 1978 39 73 79 10.1099/0022-1317-39-1-73 641533

[B66] Conroy DA Basic Atlas of Atlantic Salmon (*Salmo Salar* L*.*) Blood Cells 2006 Carrickfergus: Patterson Peddie Consulting Ltd

[B67] Lewis JM Klein G Walsh PJ Currie S Rainbow trout (*Oncorhynchus mykiss*) shift the age composition of circulating red blood cells towards a younger cohort when exposed to thermal stress J Comp Physiol B 2012 182 663 671 10.1007/s00360-012-0650-2 22322426

[B68] Krasnov A Timmerhaus G Afanasyev S Takle H Jørgensen SM Induced erythropoiesis during acute anemia in Atlantic salmon: a transcriptomic survey Gen Comp Endocrinol 2013 192 181 190 23665104 10.1016/j.ygcen.2013.04.026

[B69] Sørensen B Weber R Effects of oxygenation and the stress hormones adrenaline and cortisol on the viscosity of blood from the trout *Oncorhynchus mykiss* J Exp Biol 1995 198 953 959 9318749 10.1242/jeb.198.4.953

