# Supplementary figures and images for: Piscine orthoreovirus (PRV) infects Atlantic salmon erythrocytes
Source: Vet Res. 2014 Apr 3;45(1):35. doi: 10.1186/1297-9716-45-35 (PMC4234517; doi:10.1186/1297-9716-45-35)

Additional file 3

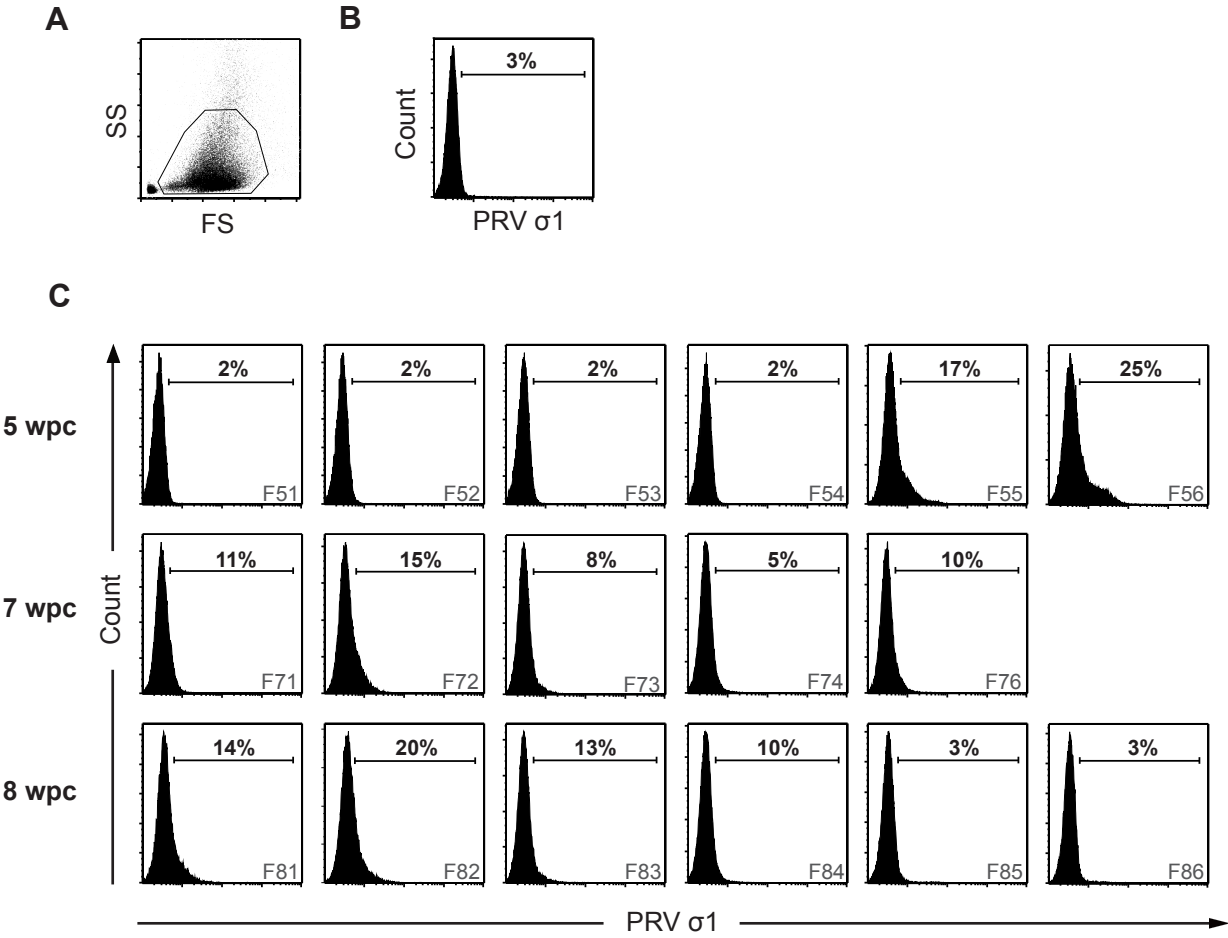

Supplement: Additional file 3 — Surface detection of PRV protein in isolated RBC from Challenge Experiment # 2 by flowcytometry.(A) Density plot showing the gating strategy for surface staining. FS, forward scatter; SS, side scatter. (B) Negative control from 0 weeks post challenge (wpc) for surface staining representing the background fluorescence. (C) Flow cytometry results from the surface staining at 5, 7 and 8 wpc. 50 000 cells were counted for each sample. Individual F75 was excluded due to technical difficulties. [file 1297-9716-45-35-S3.pdf]
